# Provision of hearing care in Pacific Island countries and territories

**DOI:** 10.2471/BLT.18.224600

**Published:** 2019-09-03

**Authors:** Peter R Thorne, Elizabeth AL Holt, Vili Nosa, Oh Chunghyeon, Sepiuta Lopati, Sione Pifeleti, Michael Maslin, Judith McCool, Berlin Kafoa

**Affiliations:** a Department of Audiology, School of Population Health, University of Auckland, Private Bag 92019, Auckland 1142, New Zealand.; b Section of Audiology, University of Auckland, Auckland, New Zealand.; c Section of Pacific Health, University of Auckland, Auckland, New Zealand.; d Department of Surgery, Colonial War Memorial Hospital, Suva, Fiji.; e Ministry of Health, Nukuʻalofa, Tonga.; f Ear. Nose and Throat Department, Ministry of Health, Apia, Samoa.; g Section of Social and Community Health, University of Auckland, Auckland, New Zealand.; h Clinical Services Program, Pacific Community, Suva, Fiji.

The World Health Organization (WHO) estimates that 466 million people globally have significant hearing loss, a figure set to double by 2050.^1^ Ear disease, such as otitis media, and hearing loss can have significant consequences on individuals, affecting communication, language development, educational opportunities and employment choices, and may also contribute to social isolation, cognitive decline and depression.^1^^,^^2^ The 2017 World Health Assembly resolution *Prevention of deafness and hearing loss* recognizes that ear disease and hearing loss are important public health issues.^3^

Prevalence of these conditions in low- and middle-income countries is higher than in high-income countries.^4^^,^^5^ WHO estimates that 75% of hearing loss in children in low- and middle-income countries is preventable through relatively low-cost and timely public health strategies^1^ such as immunization, good ear-care practices, strengthening maternal and child health programmes and screening children for otitis media. However, providing accessible hearing and ear health services to all the population remains a challenge in many of these countries because of resource constraints and competing health priorities.^6^ The 22 countries and territories (American Samoa, Cook Islands, Federated States of Micronesia, Fiji, Guam, Kiribati, Marshall Islands, Nauru, Niue, Northern Marianas, Noumea, Palau, Papua New Guinea, Pitcairn, Samoa, Solomon Islands, Tahiti, Tokelau, Tonga, Tuvalu, Vanuatu and, Wallis and Futuna) that comprise the Pacific community are located on small islands, scattered over large geographical areas. A major challenge for the Pacific islands is to develop and maintain a health workforce under such geographical challenges and resource constraints. Integrating prevention and primary hearing health services into existing health systems and focusing on broad skill development rather than standalone, specialized services, may be most appropriate.

Among other constraints, the scarce epidemiological data from Pacific Island countries and territories have hindered investment into ear and hearing health services. Yet the few available small epidemiological studies^7^^–^^9^ and model estimates^5^^,^^10^ indicate a high prevalence of acute and chronic otitis media in children in some of these countries and territories. 

Encouragingly, several small epidemiological studies are underway, such as in Kiribati, Niue, Solomon Islands and Tonga, particularly focused on children. These studies are very important to develop an overall picture of the extent and nature of ear conditions and hearing loss, but population studies are required to understand the extent of the issue and of population needs, which is essential to service planning.^11^

Although estimates show that many people have ear and hearing problems, the planning, organization and provision of relevant health services in many low- and middle-income countries, including the Pacific Islands, are relatively poor. An unpublished survey by the authors reveals sparse ear, nose and throat services and lack of formal training programmes in ear health and audiology across the region. Pacific island populations rely on visiting ear, nose and throat and audiology teams – who are mostly supported by the Royal Australasian College of Surgeons, nongovernmental organizations or charitable trusts. Yet, the region has significant workforce constraints. Fiji, with a population of close to 900 000, has two ear, nose and throat specialists; Tonga, with a population 110 000, has one; Samoa and Vanuatu have general surgeons who can provide ear, nose and throat services. One formal ear, nose and throat training programme for medical graduates is based at the University of Papua New Guinea and some formal training is offered at the Fiji National University. Trained ear, nose and throat nurses form the backbone of such services in some countries and territories; their role includes primary-care level assessments, referrals to visiting specialists and school-based screening. Stakeholders in the region are increasingly interested in training ear, nose and throat nurses as a key primary ear and hearing care workforce. However, to date no formal training programme is offered in the region, and nurses who want to specialize in this field are primarily trained by local or visiting specialists. Ear nurses, a recognized nursing specialization in New Zealand, also presents an alternative model to develop a dedicated primary ear and hearing care workforce in Pacific Island countries and territories.

Hearing services are sparse and under-resourced in the Pacific Islands, and formal audiology training is lacking. Hearing testing is typically undertaken by trained technicians or nurses. Several countries, including Fiji, Kiribati and Samoa, have built long-term partnerships with committed overseas audiologists, particularly from Australia and New Zealand. These partnerships provide visiting or volunteer in-country services, often with donated hearing aids, as well as on-the-job audiometry training. In some countries or territories, the main driver for hearing services comes from educational rather than health institutions, such as SENESE, an inclusive education support service in Samoa, and the Hilton Special School in Fiji.

Providing ear and hearing services in these countries and territories presents unique challenges because of large variations in demography. Population size is an important indicator of access to resident specialist services. For example, Niue and Tokelau have populations of approximately 1500 whereas Papua New Guinea has 8 million. All of these countries and territories have predominantly young populations, with a mean age of 22 years and a high proportion in the paediatric age group. These factors, alongside the general low priority afforded to ear, nose and throat problems in the region, may account for slow progress on development of needed services.

Over the past few years, there have been clear signs of change, with several new regional and country level initiatives reflecting the commitment, enthusiasm and support by local health professionals, civil society groups, local champions and academics from the region. Awareness of the need for ear, nose and throat, and audiology services is growing. The momentum from these small initiatives is building towards a more regional and coordinated effort supported by the Pacific Community, the main scientific and technical organization in the Pacific region, and bilateral and multilateral partners. Most notable is the leadership provided by the Clinical Services Program, funded by the Australian government and implemented by the Pacific Community. The programme takes a health systems approach to improving the planning, coordination and delivery of specialized clinical services and related workforce needs at a national level and, where sensible economies of scale exist, across the region. Support for the programme has been critical to developing a planned approach and improving awareness for ear and hearing health services at a government level. In 2015, the programme sponsored a Pacific Ear Nose and Throat Technical Advisory Group meeting where a regional plan for strengthening ear, nose and throat and audiology services in the Pacific was developed. This plan was updated^12^ and endorsed during the Heads of Health meeting in 2017, leading to implementation funding from the Australian government. The plan includes a work plan and framework for development partners to support an existing, Pacific Island-led approach to establishing ear, nose and throat and audiology services in the region. The key strategic goals include workforce capacity building, service needs evaluation, coordination of services, community engagement and the development of an ear, nose and throat and audiology hub in the South Pacific. Considerable progress has been made towards these goals. Pacific Island countries and territories have identified that a primary ear and hearing care workforce is critical to manage simple ear disease and provide ear care education to reduce the burden of ear disease and hearing loss. 

Already, primary ear and hearing care workers have been trained in several countries using WHO’s *Primary Ear and Hearing Care Training Resource* and there are now plans to establish a coordinated primary ear and hearing care workforce supported at the regional level through regular training, resource provision and clinical support. Assessment of the capacity and capability of Pacific Island countries and territories is also underway using regional surveys and WHO’s Situation Analysis Tool. 

Regional cooperation between development partners, the Pacific Community and governments is also allowing the establishment of specialist facilities, such as an Ear Health Clinic in Kiribati. International nongovernmental organizations have also begun to expand the reach of their support for population-based screening and access to hearing aids in several countries. Pacific Island countries and territories have also agreed that communication between countries can be promoted through an ear, nose and throat, and audiology hub that could provide training resources and a cloud-based repository of information, training tools, and sharing of data across the region. The hub could also provide opportunities for case conferences and clinical problem solving. A regional model is an interesting example of how countries with low resources and varying capability can work together to support each other’s service development through shared capacity building opportunities, data sharing, and support for clinical management in smaller countries.

While many challenges remain, recent local and global developments brought about by cooperation between local champions, communities and development partners present exciting opportunities to advance the delivery of services for ear and hearing health in the region. Building on the momentum generated by the endorsement of the regional plan, the timing is right for a coordinated public health approach, supported and driven by local communities, to prevent and address ear disease and hearing loss in Pacific Island countries and territories.
